# Sequential analysis for identification of byproduct from *N*-benzylation reaction: wound healing and anti-inflammatory potential of the byproduct 4-chlorobenzyl 2-((4-chlorobenzyl)amino)benzoate†

**DOI:** 10.1039/d3ra03720g

**Published:** 2023-08-30

**Authors:** Ekta Verma, Shailendra Patil, Asmita Gajbhiye

**Affiliations:** a Department of Pharmaceutical Sciences, Dr Harisingh Gour University Sagar Madhya Pradesh India agajbhiye@dhsgsu.edu.in; b Swami Vivekanand University Sagar Madhya Pradesh India

## Abstract

A very common reaction, *N*-benzylation of isatoic anhydride in the presence of sodium hydride base, produces byproducts. The yield of one of the byproducts was greater than that of the desired product; therefore, we identified the anonymous undisclosed structure of the byproduct using sequential spectroscopy methods and SC-XRD. This byproduct was found to be effective as a wound-healing and anti-inflammatory agent. The 10% formulation of byproduct and standard (nitrofurazone) showed complete wound closure with a large number of cell migrations within 16 days. Hydroxyproline contents of 5% and 10% formulations were found to be slightly increased as compared with that of the standard. The byproduct also had anti-inflammatory potential. It was effective in inhibiting COX-2, heat-induced albumin denaturation, and formalin-induced paw edema.

## Abstract 2

*N*-Benzylation of isotoic anhydride in the presence of sodium hydride base is a very common reaction. However, it produces various byproducts. Here, we will identify a major byproduct and its activities.

## Graphical abstract

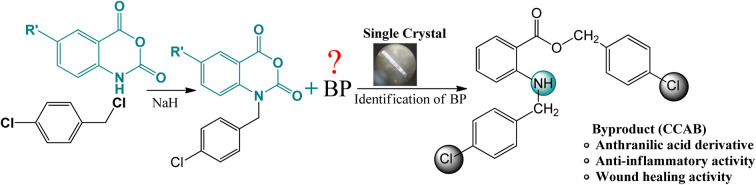

## Introduction

The formation of byproducts is very common during a chemical reaction.^1^ Many scientists are busy developing new methodologies for the optimization of reaction conditions;^2,3^ however, side reactions are sometimes more prominent than the actual main reaction. It leads to the formation of byproducts.^4^ In these cases, where the yield of the byproduct is more than the main compound, we should identify its structure as it is helpful to develop a new methodology and optimization of the reaction conditions, or sometimes these can be expedient and vendible.

Direct *N*-benzylation of isatoic anhydride is an extremely common reaction. Several bases including cesium carbonate,^5^ sodium hydride,^6–9^ sodium carbonate,^10^ potassium hydroxide,^11^ and potassium carbonate^12^ have been used for this reaction. Of these, sodium hydride is the most frequently exploited base. Herein, we identified the structure and activities of a previously unreported major byproduct, which was generated during the reaction of *N*-benzylation of isatoic anhydride in the presence of sodium hydride.

**Scheme 1:**
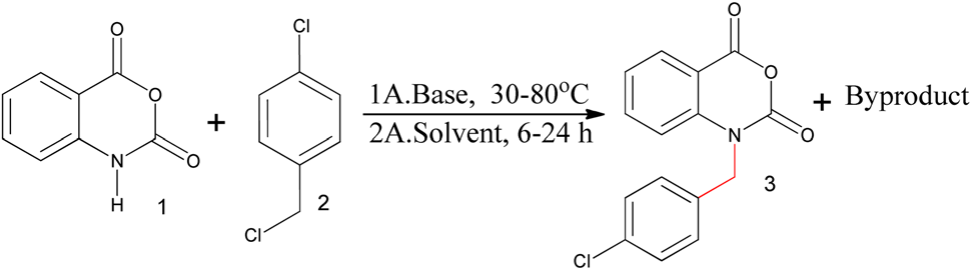
The benzylation of isatoic anhydride.

The reaction of isatoic anhydride and 4-chlorobenzyl chloride was carried out in the presence of sodium hydride base and *N*,*N*-dimethylacetamide (DMAc) as a solvent (Scheme 1). Two major compounds were formed during the reaction (Fig. S1†). One of them was the desired compound (*N*-benzylated isatoic anhydride) and the other was a byproduct. The reason for the byproduct formation was the sensitivity of the isatoic anhydride ring. The ring of isatoic anhydride is opened in the presence of a strong base (sodium hydride) under high-temperature conditions. The desired compound was found at only 48% at 30 °C,^13^ hence, we investigated the structure of the byproduct to minimize the problem of *N*-benzylation in the isatoic anhydride reaction.

Reaction conditions: 1 (1.0 mmol), 2 (1.1 mmol), base (1.0 mmol) and DMAc (1.0 ml).

## Results and discussion

The investigation of the byproduct was initiated with the optimization of the reaction conditions for the major byproduct. Different bases were investigated at varying temperatures and timeframes to boosting the percentage yield of the byproduct (Table 1, Scheme 1). The examination was initiated with weak bases such as diisopropylethylamine (DIPEA), diisopropylamine (DIPA), and cesium carbonate (Cs_2_CO_3_) at different temperatures (30 °C and 80 °C). During the reaction, CCAB was not produced with these bases. In the presence of sodium hydroxide (NaOH), many byproducts were formed at 30 °C and 80 °C. However, this base was also found to be ineffective. Furthermore, strong bases such as sodium hydride (NaH) and potassium carbonate (K_2_CO_3_) were explored. Only 31% conversion was found with the potassium carbonate base and 52% conversion with sodium hydride at 30 °C. After studying the behavior of the bases at different temperature conditions, it was concluded that the highest conversion of the CCAB can be obtained with the sodium hydride base.

**Table 1:** The investigation of bases for the CCAB formation under different temperature conditions

Entry	Base	Temp. (°C)	Time (h)	Obs. Conversion (%)
1A1	DIPEA	30	24	Nil
1A2	DIPEA	80	06	Nil
1A3	DIPA	30	24	Nil
1A4	DIPA	80	06	Nil
1A5	Cs_2_CO_3_	30	24	Nil
1A6	Cs_2_CO_3_	80	06	Nil
1A7	NaOH	30	24	Trace
1A8	NaOH	80	06	Trace
1A9	K_2_CO_3_	30	24	31
1A10	K_2_CO_3_	80	06	Trace
1A11	NaH	30	24	52
1A12	NaH	80	06	10

Consequently, the best solvent for these reaction reagents and conditions were identified (Table 2). Acetone, ethyl acetate, acetonitrile (ACN), dimethylformamide (DMF), dimethylacetamide, and tetrahydrofuran (THF) were screened as solvents (Table 4) with sodium hydride base. The product yield was obtained to be 52% with *N*,*N*-dimethylacetamide. According to this investigation, the solvents DMAc, DMF, and THF were more compatible with the synthesis of CCAB.

**Table 2:** Screening of solvents for the synthesis of CCAB

Entry	Solvent	Time (h)	Obs. conversion (%)
2A1	Acetone	24	Nil
2A2	Ethyl acetate	24	Trace
2A3	ACN	24	Trace
2A4	DMF	24	38
2A5	DMAc	24	52
2A6	THF	24	26

**Fig. 1:**
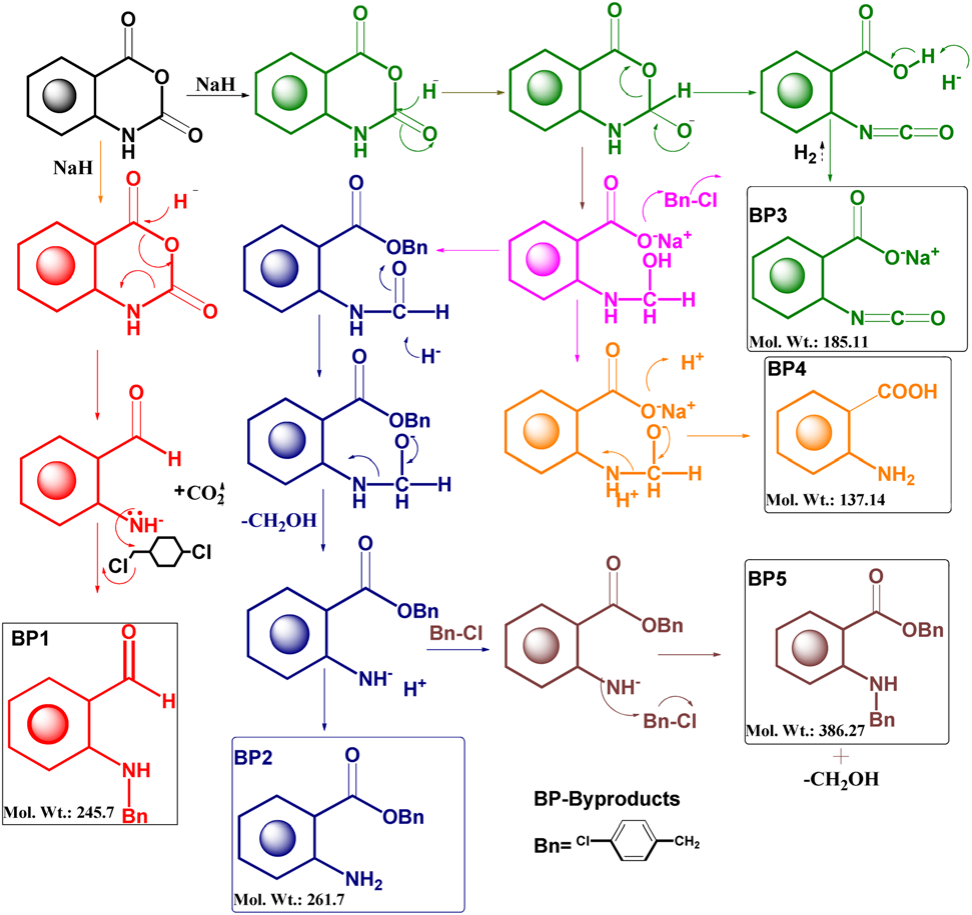
A plausible mechanism for the formation of byproducts.

After the optimization of the reaction, the crude product was purified by flash chromatography using hexane and ethyl acetate mobile phases and characterized by FTIR, ^1^H NMR, and mass spectroscopy. A plausible mechanism of the byproducts was developed for the identification of the structure^13^ (Fig. 1).

**Fig. 2:**
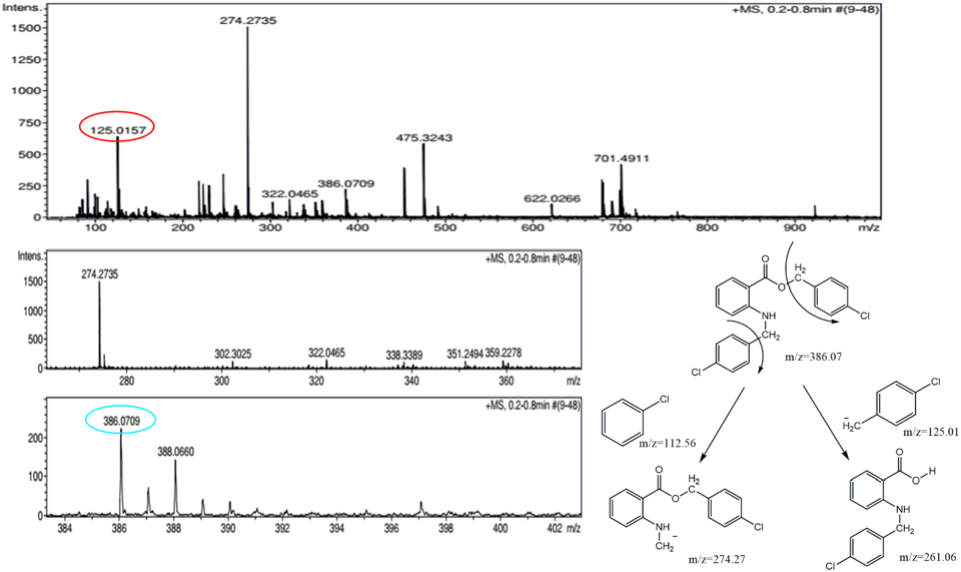
Mass spectrum of the byproduct.

The molecular weight (386.27) of one of the byproducts was correlated with the molecular ion peak of the mass spectrum. The molecular ion peak (386.07) was evident in the mass spectrum of the byproduct. The base peak was visible at 274.27, which was obtained from the breakage of the chlorobenzene moiety and other relevant fragments were also present in the mass spectrum (Fig. 2). Based on the mass spectrum, it may be inferred that the structure of the byproduct should resemble the structure of BP5.

The ^1^H-NMR spectrum showed a characteristic signal doublet at 4.393 ppm and a singlet at 5.249 ppm from the CH_2_ group.

**Fig. 3:**
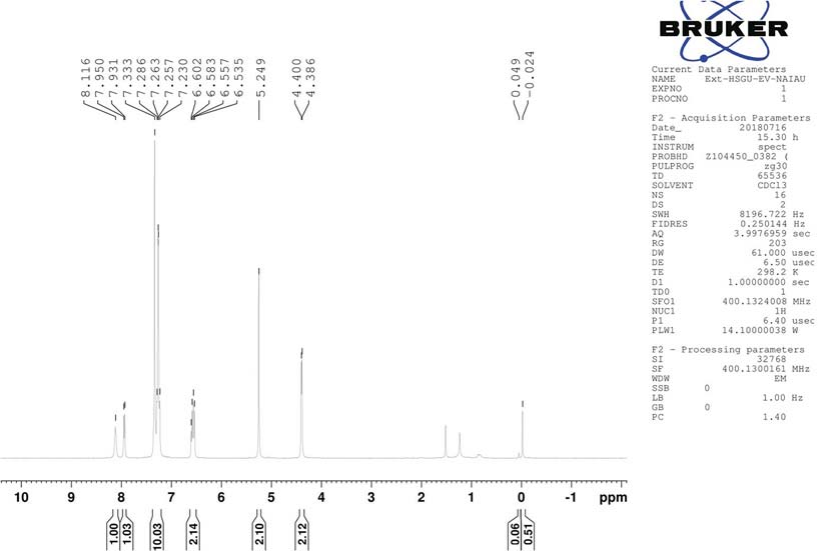
^1^H NMR spectrum of the byproduct.

**Fig. 4:**
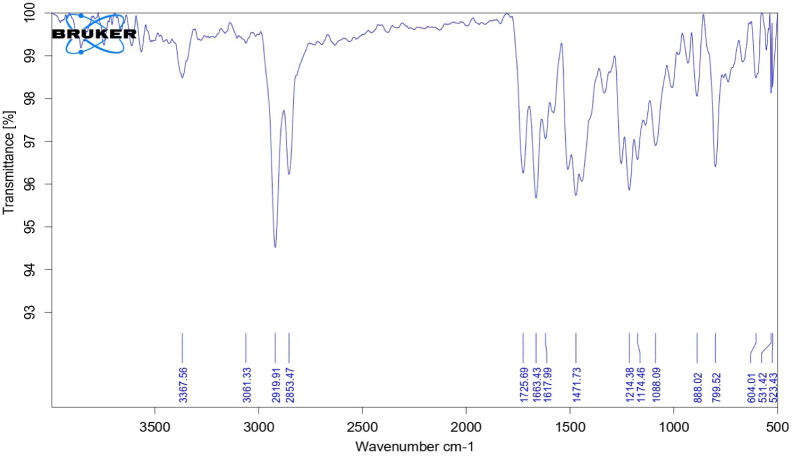
FTIR spectrum of the byproduct.

The NMR signal of the NH group was observed at 8.116 ppm and was shifted slightly upfield due to intermolecular hydrogen bonding between the –NH and –O-groups. The nearest aromatic proton of C=O appeared downfield at 7.941 ppm. The chemical shift value for other aromatic protons was in the range of 6.535–7.333 ppm (Fig. 3). On the other hand, the NH peak appeared in the IR Spectrum, and the characteristic peak of the anhydride ring disappeared (Fig. 4). Based on sequential spectroscopy analysis, it was inferred that the structure of the byproduct was similar to that of BP5.

**Fig. 5:**
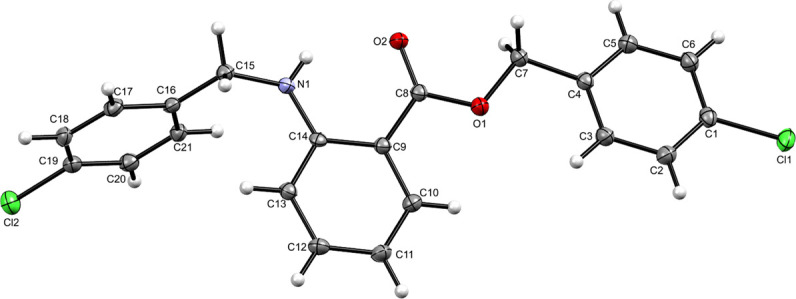
Molecular structure of the compound.

For further validation of the byproduct structure, a single crystal was grown in an anti-solvent (acetonitrile) and analyzed using a Bruker Apex-II single-crystal X-ray diffractometer equipped with a Mo Kα source and a CCD detector. The crystallography data and structure refinement details are listed in Table S1.† The refinement indicators *R*_1_ and *wR*_2_ were 0.1098 and 0.1803, respectively, which indicated the high-quality solution agreement between the calculated and observed models. The final goodness of fit (GooF) value was 0.897, which indicated that the solution was correct. The solved structure of the byproduct is shown in Fig. 5. Other structural details such as interatomic distances and bond angles, are shown in Tables S2 and S3† showing the atom-numbering scheme.

The byproduct 4-chlorobenzyl 2-((4-chlorobenzyl)amino) benzoate (CCAB) contains an anthranilic acid scaffold. Mostly anthranilic acid derivatives show anti-inflammatory activity.^14^ In CCAB, benzyl groups are substituted at the –NH and –COOH positions. It may have increased the lipophilicity of the compound and the chloro groups are attached to the para position of the benzyl group. By these substitutions, the ring becomes coplanar with the main moiety, which may be responsible to enhance the activity of wound healing by balancing the inflammatory cascade.

### The wound healing activity of CCAB

**Fig. 6:**
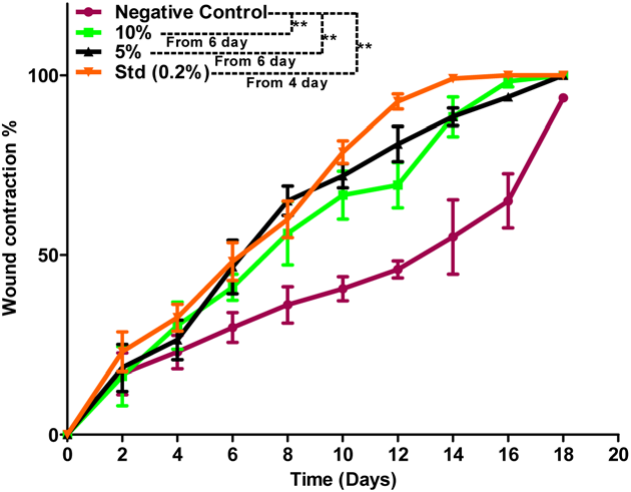
Percentage wound contraction data of the negative control, 10%, 5% formulation, and standard (0.2%) groups.

**Fig. 7:**
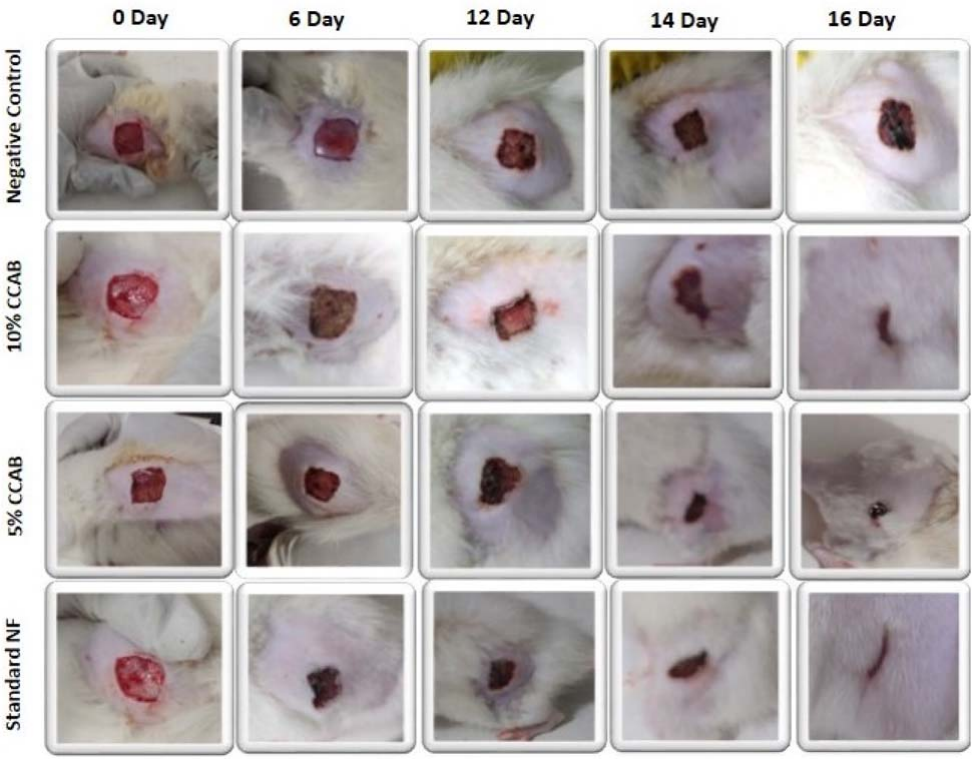
Representative images of the wound contraction with no formulation (negative control), with 5% and 10% CCAB treatment, and treatment with standard (NF).

**Fig. 8:**
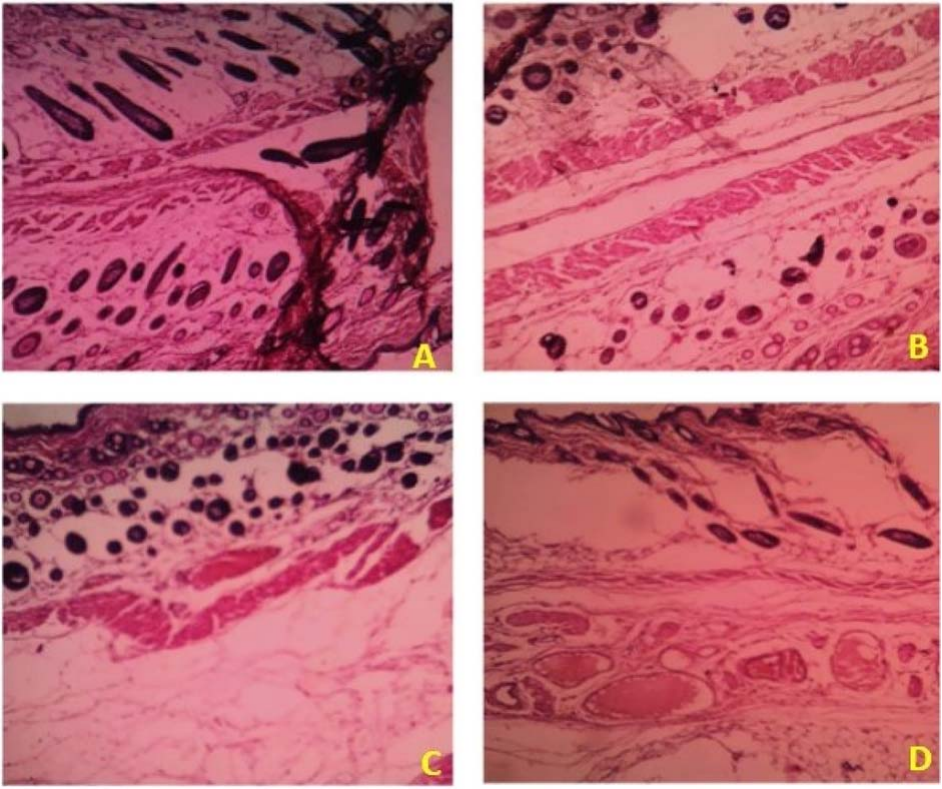
Histopathological changes in the skin wound section of (A) negative control rats showing incomplete epithelialization and damage, and minimal cellular infiltration. Skin wound section of (B) 5% treated group showing delayed and poor quality in wound healing (C) 10% treated group showing complete re-epithelialization and a large number of cell migrations. The skin wound section of (D) standard treated group showing epidermal thickening, collagen deposition, and regeneration of the skin appendages with enhanced healing.

Based on the above hypothesis, the efficacy of the byproduct (CCAB) was checked using an *in vivo* excision wound model.^15,16^ In this model, a small piece (200 mm^2^) of full-thickness skin (1 mm) was completely removed from the wound bed. The epithelialization time and the amount of granulation tissue were easily determined using this model. This study included (a) a standard (nitrofurazone ointment 0.2%) group (b) two concentrations of test compound groups (TC-5% and TC-10%) and (c) an untreatable (negative) control group. Wound size measurements (Table S4†) and percentages of wound contraction (Fig. 6) showed that the 5% (w/w) and 10% (w/w) formulations of the test compound significantly accelerated the wound area contraction compared to the negative control. A significant improvement in wound closure was initiated on day 6 with the test compound and on 4 days with the standard. Complete wound closure was observed in 10% (w/w) formulation and nitrofurazone-treated groups within 16 days (Fig. 7). The higher wound contraction rate was observed with a 10% (w/w) formulation and was also significantly compared with the standard (0.2% nitrofurazone ointment). This enhanced wound contraction by the sample might be related to the ability of the chemical ingredients to facilitate the proliferation of epithelial cells. Both, the 5% (w/w) and 10% (w/w) formulations showed wound contraction and reduced epithelialization period (Table 3). The formulation of 10% (w/w) reduced the period of epithelialization, possibly due to rapid wound contraction that shortened the distance for migrating keratinocytes. The hydroxyproline content of the compound and standard were quantified with the help of the hydroxyproline standard curve (Fig. S2†). The hydroxyproline contents of 5% and 10% formulations were found to be 5.56 ± 0.271 (μg ml^−1^) and 4.38 ± 0.178 (μg ml^−1^) (slight increase), respectively, in comparison with a standard (4.27 ± 0.178 μg ml^−1^; Table 4). To evaluate the levels of cellular infiltration and epithelialization with the various treatments, the pieces of tissue were cut, washed, and stained for histological assessment (Fig. 8). TC-10% facilitated a large number of cell migrations and complete re-epithelialization of the wound. Rats of the negative control group showed incomplete epithelialization and damage, and minimal cellular infiltration. TC-5% showed delayed and poor-quality wound healing and the standard-treated group showed epidermal thickening, collagen deposition, and regeneration of the skin appendages with enhanced healing.

**Table 3:** Epithelialization data of the negative control, 10%, 5% formulation, and standard (0.2%) groups^a^

S. No.	Group	Period of epithelialization
I	Negative control	21.67 ± 1.528
II	10%	15.67 ± 0.577**
III	5%	16.00 ± 1.00**
IV	Std 0.2%	14.33 ± 0.577**

a Values are expressed as MEAN ± SD at *n* = 6, one way ANOVA followed by Bonferroni test, *P < 0.050, **P < 0.001 and ^NS^*P* > 0.001 compared to the negative control.

**Table 4:** The hydroxyproline content of the negative control, 10%, 5% formulation, and standard (0.2%) groups^a^

S. No.	Group	Hydroxyproline content (μg ml^−1^)
I	Negative control	5.95 ± 0.268
II	10%	4.38 ± 0.178**
III	5%	5.56 ± 0.271**
IV	Std 0.2%	4.27 ± 0.178**

a Values are expressed as MEAN ± SD at *n* = 6, one way ANOVA followed by Bonferroni test, *P < 0.050, **P < 0.001 and ^NS^*P* > 0.001 compared to the negative control.

Values are expressed as MEAN ± SD at *n* = 6, one way ANOVA followed by Bonferroni test, *P < 0.050, **P < 0.001 and ^NS^*P* > 0.001 compared to the negative control.

### Anti-inflammatory activity

To assess the efficacy of CCAB, anti-inflammatory activities were determined by *in vitro* and *in vivo* models.

### Albumin denaturation

**Fig. 9:**
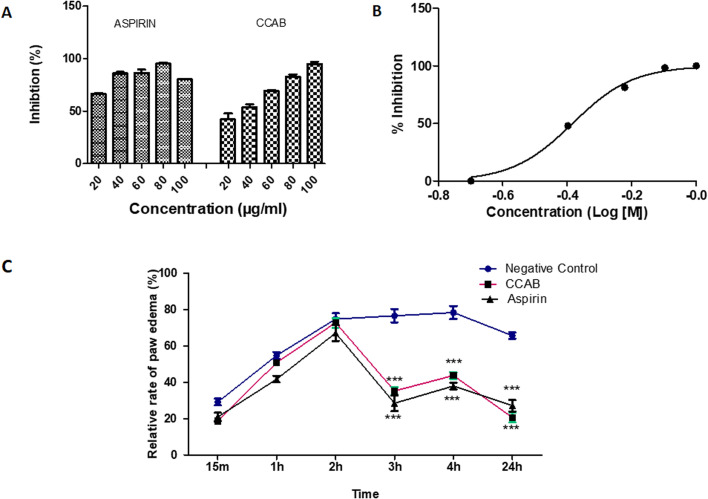
(A) Effect of compound (CCAB) and standard (Aspirin) on heat-induced protein denaturation. (B) Log dose–response curves for CCAB. (C) Graphical presentation of the effects of compound (CCAB) and standard (Aspirin) on formalin-induced paw edema at 15 min, 1, 2, 3, 4, and 24 hours.

The newly synthesized compound (CCAB) was found to be effective in inhibiting heat-induced albumin denaturation. The percentage inhibition of protein denaturation was concentration-dependent (Fig. 9A). The maximum inhibition of the CCAB was observed at 100 μg ml^−1^ with 95.4% and that of aspirin was at 80 μg ml^−1^ with 95.4%.

### COX-2 enzyme assay

The synthesized compound CCAB significantly inhibited the COX 2 enzyme in the micro-molar range. The IC_50_ value of CAAB was 0.41 μM (Fig. 9B).

### Formalin-induced rat paw edema

Results of the acute anti-inflammatory test by formalin-induced paw edema revealed that both the test and standard were able to significantly reduce paw volume in comparison with the control group at 3, 4, and 24 h (Fig. 9C). The paw edema in control rats increased along with time. The mean% swelling of the negative control group was 78.4% at 4 h. CCAB and standard (Aspirin) at the dose of 300 mg per kg bw showed significantly decreased paw edema, 35.3% and 28.5% of swelling, respectively, at 4 h.

## Conclusion

The structure of the byproduct CCAB was successfully identified with the help of sequential spectroscopy methods (IR, NMR, and MASS) and validated by a single crystallographic technique. The 10% formulation of CCAB showed complete wound closure with a large number of cell migrations within 16 days, similar to a standard (nitrofurazone). This formulation also significantly reduced the period of epithelialization, possibly due to rapid wound contraction, which shortened the distance for migrating keratinocytes. The hydroxyproline contents in 5% and 10% formulations were found to be 5.56 ± 0.271 (μg ml^−1^) and 4.38 ± 0.178 (μg ml^−1^) (slight increase), respectively, in comparison with a standard (4.27 ± 0.178 μg ml^−1^). The byproduct CCAB also had anti-inflammatory potential. It has been found to be effective in inhibiting heat-induced albumin denaturation and formalin-induced paw edema. The maximum inhibition of the compound was observed at 100 μg ml^−1^ with 95.1% in the case of heat-induced albumin denaturation. The mean% swelling of the negative control group rats was found to be 78.4% in 4 h. CCAB and standard (Aspirin) at the dose of 300 mg per kg bw, significantly decreased the paw edema by 35.3 and 28.5% of swelling, respectively, in 4 h. On the basis of these studies, the byproduct (CCAB) proved to be a lead compound that may be used in the treatment of wound healing by controlling excessive inflammation.

## Experimental

### Material and methods

All chemical, solvent, and TLC plates were purchased from HiMedia, Spectrochem, Sigma-Aldrich, and Merck. Reactions were performed in oven-dried glassware and solvents were dried using distillation followed by the addition of 3 Å activated (250 °C for 2 h) molecular sieves. The progress of the reactions was followed using silica gel G TLC plates with F-254 and spots were visualized under a UV chamber at 254 and 365 nm. The purification of the compounds was also performed by flash chromatography (EPCLC AL-580S & Yamazen Corporation) on silica gel with hexane and ethyl acetate as a mobile phase. The melting points of the synthesized compounds were recorded using Thiele's tube apparatus. The infrared spectra of the compounds were recorded using FT-IR (Bruker Alfa, ECO-ATR). Proton and carbon-13 nuclear magnetic resonance spectra were recorded using a Bruker DX 400 MHz spectrometer. DMSOd_6_ was used as a solvent. In ^1^H NMR, chemical shifts were reported in parts per million and tetramethylsilane was used as an internal standard. HRMS was recorded on a Bruker Micro TOF QII instrument. Single-crystal X-ray diffraction data of the monoclinic form of the compound was collected on a Smart Apex-II single-crystal X-ray diffractometer. The crystal was maintained at 296.15 K during data collection. Using Olex2, the structure was solved with the XS structure solution program using direct methods and refined with the ShelXL refinement package using least squares minimization.

### Optimized procedure of synthesis of CCAB

Sodium hydride (57% in mineral oil, 4.2 g, 0.10 mol) was added to the solution of isatoic anhydride (16.3 g, 0.10 mol) in *N*,*N*-dimethylacetamide (200 ml) with continuous stirring at 30 °C. After 1 hour, 4-chlorobenzyl chloride (17.7 g, 0.11 mol) was added to the reaction mixture. The reaction mixture was stirred for 24 hours. The reaction mixture was poured into the crushed ice. The formed precipitate was filtered and washed with cold water and dried. The crude compound was purified by column chromatography using hexane and ethyl acetate solvent.^13^ The pure compound was obtained as a white crystalline solid (yield 52%; m.p. 89–90 °C, *R*_f_ 0.72).

### Crystallization procedure

The product was dissolved in an acetonitrile anti-solvent with the help of a thermo-control hot plate. The vials were tightly closed and slowly cooled to room temperature (298 K). After 2 days, good-quality crystals were obtained and a suitable single crystal was selected under an optical microscope for the X-ray diffraction study. The data of single crystal were collected on a Bruker APEX2 single crystal X-ray diffractometer equipped with a Mo Kα source and a CCD detector. The data was integrated and scaled using the Bruker suite of programs. A crystal was attached to a thin glass fiber with epoxy glue and mounted on a goniometer head and the data were collected at 100 degrees per K using an Oxford Cryostream 800 Plus cryostat. The structures were solved by direct methods and refined by full-matrix least-squares on F2 using SHELX-2014. All non-hydrogen atoms were refined anisotropically and all the hydrogen atoms were placed using calculated positions and riding models.

## Pharmacological activity

### Wound healing activity (*in vivo*): an excision wound model

Healthy albino Wistar rats strain of either sex weighing 150 to 250 g were selected for the study. The animals were kept under 12 : 12 h day and light schedules with temperatures between 18 to 20 °C. They were housed in large, spacious hygienic cages during the experimental period. Animals were allowed free access to water and a standard pellet diet up to the end of the study.

### Preparation of the sample

The compound was dissolved in carboxymethylcellulose (CMC; 1%) to prepare the formulations (5% and 10%).

### Experiment

The animals were anesthetized with slight vapor inhalation of diethyl ether, and the hair was removed from the dorsal thoracic region. Excision wounds of 200 mm^2^ size and 1 mm depth were made by cutting out pieces of skin from the shaved area. The entire wound was left open. The animals were closely observed for any infection and those that showed any sign of infection were separated, excluded from the study, and replaced. Then, the samples (5% and 10%) and standard nitrofurazone 0.2% ointment were applied every day to the specified groups for 20 days. Wound areas were measured on days 0, 2, 4, 6, 8, 10, 12, 14, 16, 18, and 20 for all groups, using a transparency sheet and a permanent marker. The day the scar fell off, after the wound formation without any residual raw wound, was considered the day of epithelialization. This model was used to monitor the rate of wound contraction and epithelialization.

The percentage of wound contraction was calculated as follows
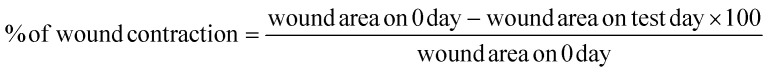


The falling of the scab from around the wound was taken as the end point of complete epithelialization and the total days required for this were taken as the period of epithelialization. From the healed wound, a tissue specimen sample was isolated from each group of rats for histopathological examination.

### Epithelialization period

It was evaluated by noting the number of days required for Escher to fall off from the wound surface, exclusive of leaving a raw wound behind.

### Hydroxyproline estimation

0.1 ml of hydrolysate sample was pipetted out into a clean test tube, and the volume was made up to 0.5 ml with distilled water. From the stock solution of standard hydroxyproline, 1.6 ml was taken and diluted up to 100 ml. From this, 0.5 (8 μg) was pipetted out into a clean test tube. To this, 1 ml each of 2.5 N NaOH, 0.01 M CuSO_4_, and 6% H_2_O_2_ were added. Immediately, the tubes were placed in a water bath at 80 °C for 16 minutes and then cooled for 5 minutes. To this, 2 ml of freshly prepared 5% solution of *para*-dimethylamino-benzaldehyde in *n*-propanol, and 4 ml of 3 N H_2_SO_4_ were added. Test tubes were once again placed in a hot water bath at 80 °C for 15 minutes and then cooled for 5 minutes. The optical density (O.D.) of the pink color of these test samples was compared with that of standard hydroxyproline of known concentration at 540 nm using a UV-vis spectrophotometer for the estimation of hydroxyproline.

### Anti-inflammatory activity

Anti-inflammation activity was evaluated by albumin denaturation,^17^ the Elisa kit for cyclooxygenase-2 (ref. 18), and formalin-induced rat paw edema methods.^19,20^

### Albumin denaturation assay (*in vitro*)

Different dilutions (20, 40, 60, 80, and 100 μg ml^−1^) of the test compound were prepared from the stock solution (1000 μg ml^−1^) using 1% DMSO. The reaction mixture for the albumin denaturation assay was composed of the test sample, 1% BSA, and PBS (phosphate buffer saline; pH 7.4). The reaction mixture was incubated at 37 °C for 15 minutes followed by incubating the same at 70 °C for 10 minutes to cause albumin denaturation by pyrolysis. After 10 minutes of incubation, the reaction mixture was allowed to cool and absorbance was measured spectroscopically at 660 nm. Aspirin was used as the reference standard for the study. The percentage inhibition and IC_50_ of the compounds under study were calculated.

### COX-2 enzyme assay

The COX-2 assay was performed using an ELISA kit for cyclooxygenase-2 (SEA699Hu).

### Formalin-induced rat paw edema (*in vivo*)

Female Wistar albino rats weighing 100–150 g were purchased and they were acclimatized under-maintained laboratory conditions of humidity (50%), temperature (25 ± 2 °C), and 12 hours dark/light cycle. The animals were segregated into three groups (*n* = 3). The first group served as a negative control (physiological saline, 5 ml kg^−1^), the second group received a test compound (300 mg kg^−1^), and the third group received aspirin (300 mg kg^−1^) as a reference standard. The animals were subjected to overnight fasting. Edema was induced with a 0.1 ml injection of formalin solution (2.5%) into the plantar region of the left hind paw. After 1 hour of drug administration, paw thickness was measured hourly at 0, 1, 2, 3, and 4 hours after the formalin injection. The anti-inflammatory potency of the test compound to suppress paw inflammation was expressed as a percentage inhibition of paw edema and calculated according to the followingRelative paw edema = (*v*_2_ − *v*_1_/*v*_1_) × 100where, *v*_1_ is the paw thickness of the control animal, *v*_2_ is the paw thickness of the test compound's animal at different time points.

## Conflicts of interest

There are no conflicts to declare.

## Supplementary Material

RA-013-D3RA03720G-s001

RA-013-D3RA03720G-s002
