# Supplementary material for: Sequential analysis for identification of byproduct from N-benzylation reaction: wound healing and anti-inflammatory potential of the byproduct 4-chlorobenzyl 2-((4-chlorobenzyl)amino)benzoate
Source: RSC Adv. 2023 Aug 30;13(37):25904–11. doi: 10.1039/d3ra03720g (PMC10466176; doi:10.1039/d3ra03720g)
Supplement: RA-013-D3RA03720G-s001 [file RA-013-D3RA03720G-s001.pdf]

Electronic Supplementary Material (ESI) for RSC Advances.
This journal is © The Royal Society of Chemistry 2023

Supporting Information of

Sequential analysis for identification of byproduct from N-benzylation reaction: Wound healing and anti-inflammatory potential in byproduct 4-chlorobenzyl 2-((4-chlorobenzyl)amino) benzoate

Sequential analysis for identification of byproduct from N-benzylation reaction: Wound healing and anti-inflammatory potential in byproduct 4-chlorobenzyl 2-((4-chlorobenzyl)amino) benzoate

Ekta Verma[1], Shailendra Patil[2], and Asmita Gajbhiye[1]*

[1]Department of Pharmaceutical Sciences, Dr. Harisingh Gour University, Sagar, Madhya Pradesh, India

[2]Swami Vivekanand University, Sagar, Madhya Pradesh, India

*Corresponding author: E-mail: drasmitagajbhiye27@gmail.com

Table of Contents

TLC* image of crude compound (NaH, 30°C)........S3

Standard curve for Hydroxyproline........S4

Crystal data and structure refinement for CCAB........S5

Interatomic distances (Å) of 4-chlorobenzyl 2-((4-chlorobenzyl)amino) benzoate........S6

Bond Angles (degree) of 4-chlorobenzyl 2-((4-chlorobenzyl)amino)benzoate........S6

Wound Contraction (mm) data of negative control, 10%, 5% formulation and standard (0.2%) groups........S8

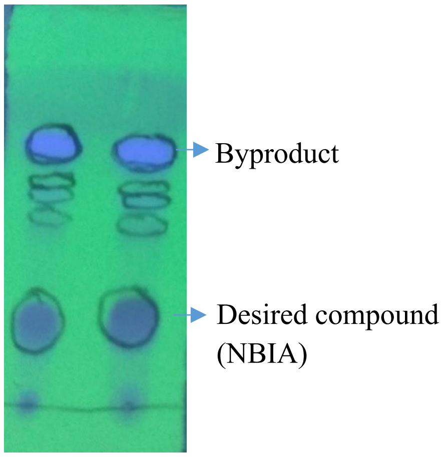

Figure S1. TLC* image of crude compound (NaH, 30°C)

NBIA- N-benzylated isatoic anhydride

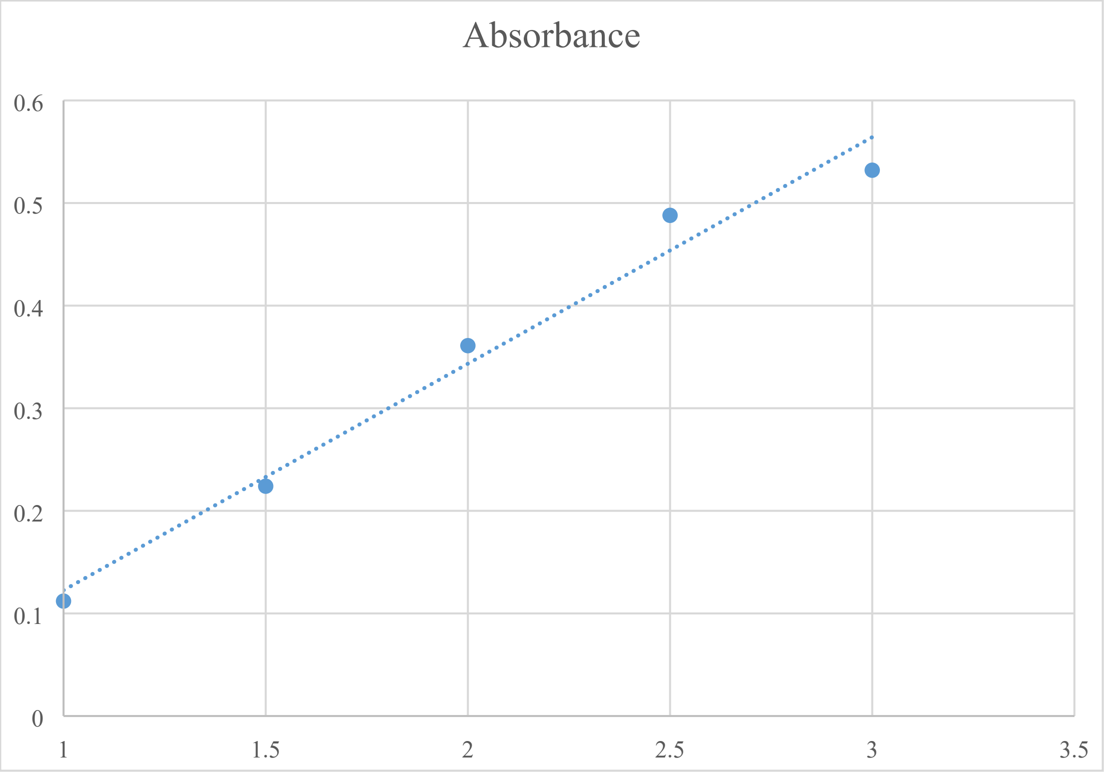

Figure S2. Standard curve for Hydroxyproline

Table S1. Crystal data and structure refinement for CCAB.

Identification code	M
Empirical formula	$C_{21}H_{17}Cl_2NO_2$
Formula weight	386.26
Temperature/K	296.15
Crystal system	monoclinic
Space group	$P2_1/c$
a/Å	15.168(8)
b/Å	5.683(3)
c/Å	21.306(11)
α/°	90
β/°	100.272(9)
γ/°	90
Volume/Å^3	1807.1(16)
Z	4
ρ_{calc}g/cm^3	1.420
μ/mm^{-1}	0.375
F(000)	800.0
Crystal size/mm^3	0.36 × 0.22 × 0.2
Radiation	MoKα (λ = 0.71073)
2Θ range for data collection/°	8.034 to 58.276
Index ranges	$-16 \leq h \leq 20$, $-7 \leq k \leq 7$, $-28 \leq l \leq 23$
Reflections collected	7043
Independent reflections	4330 [R_{int} = 0.0496, R_{sigma} = 0.0887]
Data/restraints/parameters	4330/0/239
Goodness-of-fit on F^2	0.897
Final R indexes [I>=2σ (I)]	R_1 = 0.0598, wR_2 = 0.1454
Final R indexes [all data]	R_1 = 0.1098, wR_2 = 0.1803
Largest diff. peak/hole / e Å^{-3}	0.30/-0.40

Table S2 .Interatomic distances (Å) of 4-chlorobenzyl 2-((4-chlorobenzyl)amino)

benzoate

Atom	Atom	Length/Å	Atom	Atom	Length/Å
**Cl1**	C1	1.754(3)	C8	C9	1.470(4)
**Cl2**	C19	1.737(3)	C9	C10	1.411(4)
**O1**	C7	1.439(4)	C9	C14	1.421(4)
**O1**	C8	1.345(3)	C10	C11	1.377(4)
**O2**	C8	1.223(3)	C11	C12	1.392(4)
**N1**	C14	1.373(4)	C12	C13	1.380(4)
**N1**	C15	1.451(4)	C13	C14	1.404(4)
**C1**	C2	1.376(4)	C15	C16	1.511(4)
**C1**	C6	1.380(4)	C16	C17	1.396(4)
**C2**	C3	1.389(4)	C16	C21	1.393(4)
**C3**	C4	1.384(4)	C17	C18	1.388(5)
**C4**	C5	1.389(4)	C18	C19	1.386(4)
**C4**	C7	1.508(4)	C19	C20	1.373(4)
**C5**	C6	1.380(4)	C20	C21	1.386(4)

Table S3. Bond Angles (degree) of 4-chlorobenzyl 2-((4-chlorobenzyl)amino)benzoate

Atom	Atom	Atom	Angle/°
**C8**	O1	C7	116.2(2)
**C14**	N1	C15	122.7(3)
**C2**	C1	Cl1	119.3(2)
**C2**	C1	C6	121.6(3)
**C6**	C1	Cl1	119.0(2)
**C1**	C2	C3	118.7(3)
**C4**	C3	C2	121.0(3)
**C3**	C4	C5	118.8(3)
**C3**	C4	C7	123.6(3)
**C5**	C4	C7	117.6(3)
**C6**	C5	C4	121.0(3)
**C5**	C6	C1	118.8(3)
**O1**	C7	C4	109.1(2)
**O1**	C8	C9	112.4(2)
**O2**	C8	O1	121.6(3)
**O2**	C8	C9	125.9(3)
**C10**	C9	C8	119.3(3)
**C10**	C9	C14	119.1(3)
**C14**	C9	C8	121.6(3)
**C11**	C10	C9	121.9(3)
**C10**	C11	C12	118.7(3)
**C13**	C12	C11	120.9(3)
**C12**	C13	C14	121.6(3)
**N1**	C14	C9	121.4(3)
**N1**	C14	C13	120.8(3)
**C13**	C14	C9	117.8(3)
**N1**	C15	C16	115.0(3)
**C17**	C16	C15	119.5(3)
**C21**	C16	C15	122.7(3)
**C21**	C16	C17	117.9(3)
**C18**	C17	C16	121.1(3)

**C19**	C18	C17	119.3(3)
**C18**	C19	Cl2	119.3(2)
**C20**	C19	Cl2	119.8(3)
**C20**	C19	C18	120.9(3)
**C19**	C20	C21	119.4(3)
**C20**	C21	C16	121.5(3)

Table S4. Wound Contraction (mm) data of negative control, 10%, 5% formulation and standard (0.2%) groups

S. No.	Group	Time (Days)										
		**0**	**2**	**4**	**6**	**8**	**10**	**12**	**14**	**16**	**18**	**20**
I	**Negative control**	214.83±2.041	178.50±11.467	165.50±9.586	150.83±8.424	137.33±10.708	127.67±7.062	116±4.336	94.67±21.979	75.00±16.432	13.50±1.871	4.00±6.229
II	**10 %**	209.17±9.326^{NS}	174.83±13.394^{NS}	145.67±13.736^{NS}	123.33±8.454**	91.83±18.798**	69.97±14.787**	64.17±15.497**	21.83±9.152**	2.00±1.897**	0±0^{NS}	0±0^{NS}
III	**5%**	211.17±6.795^{NS}	177.67±15.384^{NS}	155.83±15.381^{NS}	112.83±17.759**	73.67±9.522**	59.00±7.925**	40.33±10.727**	24.33±5.317**	12.67±2.160**	0±0^{NS}	0±0^{NS}
IV	**Std 0.2%**	213.33±1.966^{NS}	164.17±12.205^{NS}	144.00±8.764**	110.50±10.56**	85.50±10.858**	45.50±6.745**	15.50±4.370**	2.00±2.530**	0±0^{NS}	0±0^{NS}	0±0^{NS}

Values are expressed as MEAN±SD at n=6, One way ANOVA followed by Bonferroni test, *P<0.050, **P<0.001 and ^{NS}P>0.001 compared to the negative control
